# Comparision effects of solifenacin, darifenacin, propiverine on ocular parameters in eyes: A prospective study

**DOI:** 10.1590/S1677-5538.IBJU.2019.0094

**Published:** 2020-01-10

**Authors:** Mahmut Taha Ölçücü, Kerem Teke, Kadir Yildirim, Mesut Toğaç, Burcu Işık, Yusuf Cem Yilmaz

**Affiliations:** 1 Department of Urology Agri State Hospital Agri Turkey Department of Urology, Agri State Hospital, Agri, Turkey;; 2 Department of Urology Elaziğ Education and Research Hospital Elazig Turkey Department of Urology, Elaziğ Education and Research Hospital, Elazig, Turkey;; 3 Department of Ophthalmology Agri State Hospital Agri Turkey Department of Ophthalmology, Agri State Hospital, Agri, Turkey

**Keywords:** Pressure, Urinary Bladder, Overactive, Anisocoria

## Abstract

**Objective:**

To evaluate the effects of solifenacin, darifenacin, and propiverine on nasal-, subfoveal-, temporal choroidal thicknesses (NCT, SFCT, TCT), intraocular pressure (IOP) and pupil diameter (PD).

**Materials and Methods:**

Patients with overactive bladder (OAB) diagnosed according to The International Continence Society were administered with solifenacin, darifenacin or propiverine on a daily basis between November 2017 and May 2018. NCT, SFCT, TCT, IOP, and PD of these patients were measured and compared as initial, fourth and twelfth weeks.

**Results:**

A total of 165 patients (330 eyes) with OAB were evaluated. Solifenacin (n=140) significantly reduced IOP from 17.30±2.72 mmHg to 16.67±2.56 mmHg (p=0.006) and 16.57±2.41 mmHg (p=0.002), at the fourth and twelfth weeks, respectively. Darifenacin (n=110) significantly reduced NCT from 258.70±23.96 μm to 257.51±22.66 μm (p=0.002) and 255.36±19.69 μm (p=0.038), at the fourth and twelfth weeks, respectively. Propiverine (n=80) significantly increased PD from 4.04±0.48 mm to 4.08±0.44 mm (p=0.009) and 4.09±0.45 mm (p=0.001), at the fourth and twelfth weeks, respectively.

**Conclusion:**

These findings can help to decide appropriate anticholinergic drug choice in OAB patients. We finally suggest further well-designed randomized prospective studies with a larger population to evaluate the anticholinergic-related complications in eyes.

## INTRODUCTION

Both sexes are affected by an overactive bladder (OAB), which is a common disorder with a reported prevalence between 11.8% and 16.9%. Symptoms of OAB include urgency, frequent urination, and nocturia with or without urge incontinence (1-3). Lifestyle modifications and behavioral therapies are the first steps taken into treatment, however, medical therapy constitutes the mainstay method (4). Oral anticholinergics are the first-line drugs in medical treatment (5). Due to various settlements of cholinergic (muscarinic) receptors (M^1^-M5) in the body, the side effects of anticholinergics emerge in accordance with this (6). These drugs can cause side effects such as dry mouth, constipation, nausea, vomiting, headache, and confusion (7, 8).

There are extensive M receptors in iris sphincter muscle, ciliary muscle and trabecular cells (9-11). Iris sphincter muscle and ciliary muscles are relevant with accommodation and changing of pupil diameter (PD) (12, 13). M3 receptors are most common cholinergic receptors than the other types in iris sphincter and ciliary muscle cells (10, 14). Trabecular cells control intraocular pressure (IOP) by changing the aqueous humor drainage (15). Blockage of these receptors may lead to blurred vision, dry eyes, and increased IOP in angle-closure glaucoma (16, 17). Choroid is one of the most vascularised areas in the body, allowing outer retina to become vascularised (18). Increased choroidal thickness (CT) can cause haemorrhage and exudation. Reducing this thickness can decrease blood flow of retina (18). Age-specific (haemorrhagic and exudated) choroidal thickening is known to be a poor prognostic factor in age-related macular degeneration. It is reported that choroidal layer contains smooth muscles with intense cholinergic innervations (18, 19).

In this context, since anticholinergic drugs may affect ocular M receptors, we herein aimed to evaluate the effects of solifenacin, darifenacin, and propiverine on nasal-, subfoveal-, temporal CTs (NCT, SFCT, TCT), IOP and PD in the eyes.

## MATERIALS AND METHODS

**Figure 1:**
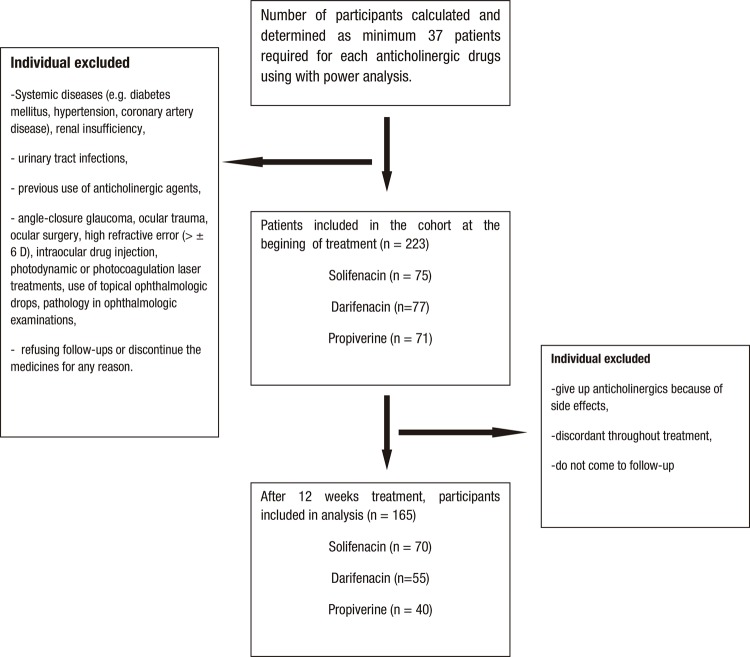
Flow-chart of the our prospective cohort study.

The study was designed prospectively non-randomized in accordance with the Helsinki Declaration after approval of Erzurum Atatürk University Medical Faculty Ethics Committee (Approval Number: B.30.2.ATA.0.01.00/128). Patients who were diagnosed with OAB in urology clinic between November 2017 and May 2018 were included and referred to ophthalmologic clinic after receiving the informed consents from patients. The diagnosis of OAB was made according to the description described earlier by the International Continence Society (ICS) (20). Informed consent forms were obtained from patients who wanted to participate in the study. The costs of the drugs were paid by the patient’s own health insurance. As a result of detailed urological and ophthalmological evaluations, patients with histories of any systemic diseases (e.g. diabetes mellitus, hypertension, coronary artery disease), renal insufficiency, urinary tract infections, previous use of anticholinergic agents, angle-closure glaucoma, ocular trauma, ocular surgery, high refractive error (>±6D), intraocular drug injection, photodynamic or photocoagulation laser treatments, use of topical ophthalmologic drops, pathology in ophthalmologic examinations and refusing follow-ups or discontinue the medicines for any reason were excluded from the study (Figure-1). No patient gave up on OAB treatment because of ocular findings or ocular side effects.

Patients with OAB were administered with 5mg solifenacin 1x1, 7.5mg darifenacin 1x1 or 30mg sustained-release propiverine 1x1 on a daily basis. When we analyzed retrospectively, we found that the most preferred anticholinergics were solifenacin, darifenacin, propiverine, oxybutynin, and trospium, respectively in our clinic. Hence we aimed to compare the eyes of patients who were treated with most preferred three anticholinergics (solifenacin, darifenacin, propiverine) for OAB treatment. As known, the effects of anticholinergics are similar. But more adverse effects can be observed with some anticholinergics than others. Two urologists (MTO and KT) distributed the anticholinergics in a regular order to the patients with OAB. We did not make any randomization in this study. We tried to keep equally the number of groups. Some factors affected the number of participants who have finished the study among groups. Those factors included: patients who gave up the anticholinergic because of adverse effects, did not come to follow-up, and were discordant throughout treatment. In method, we have specified those factors as the excluded criteria described above. We observed more adverse effects in propiverine group. And it affected the number of propiverine group. The ophthalmologists who examined the patients did not know which anticholinergics were used by the patients. SFCT, NCT, TCT, IOPs, and PDs in eyes of the patients in the fourth and twelfth weeks were evaluated by ophthalmologists. CT was measured at 500-micron intervals of the section from 1mm nasal and temporal to the subfoveal center via an optical coherence tomography device (Topcon 3d Oct-2000 Fa Plus, Topcon Inc., Japan). IOP was measured with a computerized tonometer (Topcon Ct-800, Topcon Inc., Japan), and PD was measured by an autorefractometer (Topcon Kr-800, Topcon Inc., Japan).

When we assigned the statistical power as 0.8, the alpha as 0.05, effect size as 0.3 and group size as 3 the power analysis has shown minimum required sample size as 111. So a minimum of 37 patients were required for each anticholinergic drug. The mean values of the measurements for both eyes were recorded as data for each patient. The results were statistically analyzed with SPSS 15.0. Normality test was used to determine the pretreatment measurements among solifenacin, darifenacin, propiverine groups. Due to the data is normally distributed, ANOVA test was used to compare independent variables regarding those three groups. For the ages of the three groups, the Kruskal Wallis test was used after the descriptive statistics, and two samples paired t-test was used to compare the values measured in the fourth and twelfth weeks for the same individuals depending on the medicines used. A value of p <0.05 was deemed statistically significant.

## RESULTS

One hundred sixty-five patients (330 eyes) who have completed 12 weeks follow-up received solifenacin (n=70), darifenacin (n=55), and propiverine (n=40) (Figure-1). A hundred eighteen (71.5%) patients were female and 47 (28.5%) were male. Female and male ratio for the solifenacin, darifenacin and propiverine groups were 59 (84.3%) vs. 11 (15.7%), 30 (54.5%) vs. 25 (45.5%), and 29 (72.5%) vs. 11 (27.5%), respectively. Total mean age was 50.10±13.17 years (ranging from 19 to 81) and observed as 49.37±13.17 (ranging from 19 to 76) in the solifenacin group, as 50.49±12.50 (ranging from 21 to 75) in the darifenacin group, and as 50.87±14.31 (ranging from 29 to 81) in the propiverine group. There was no significant difference between the mean age of the groups (p=0.848). No statistically differences were determined between baseline pretreatment measurement of SCFT (p=0.587), NCT (p=0.430), TCT (p=0.365) and PD (p=0.960) among solifenacin, darifenacin, propiverine groups. However, baseline pretreatment values of IOP (p=0.010) were statistically difference among those three groups (Table-1).

**Table 1:** The pretreatment results of descriptive analysis regarding to three anticholinergic groups are presented.

	Solifenacin (n=70)	Darifenacin (n=55)	Propiverine (n=40)	P
**Gender**				
Female (%)	59 (84.5%)	30 (54.5%)	29 (72.5%)	-
Male (%)	11 (15.5%)	25 (45.5%)	11 (27.5%)	
Age (mean ± SD)	49.37±13.17	50.49±12.50	50.87±14.31	0.848
Subfoveal choroidal thickness (μm) (mean ± SD)	253.52±26.72	258.66±33.08	258.80±38.08	0.587
Nasal choroidal thickness (μm) (mean ± SD)	252.48±24.64	258.70±23.96	256.17±33.34	0.430
Temporal choroidal thickness (μm) (mean ± SD)	251.30±29.43	258.08±22.98	251.47±33.53	0.365
Intraocular pressure (mmHg) (mean ± SD)	17.30±2.72	17.05±3.40	15.50±3.02	0.010*
Pupil diameter (mm) (mean ± SD)	4.06±0.53	4.03±0.43	4.04±0.48	0.960

The Kruskal Wallis test was used to compare the three treatment groups by age.

To compare independent variables regarding to those three groups, the ANOVA test was used.

* p<0.05 value was accepted for the statistical difference.

### Choroidal Thickness

Subfoveal Choroidal Thickness: No statistically significant differences were observed between the pretreatment and fourth week or between the pretreatment and the twelfth week for SCFT in the solifenacin-, darifenacin-, and propiverine-treated groups (p >0.05) (Table-2). Nasal Choroidal Thickness: The mean value of NCT slightly but statistically increased at fourth week in solifenacin group (p=0.042).. However, rising NCT regressed to approximately pretreatment levels at the twelfth week in these patients (p=0.849). The darifenacin treatment gradually reduced the mean of NCT at the fourth and twelfth weeks compared to pretreatment levels. These decreases were significantly different at the fourth (p=0.002) and twelfth weeks (p=0.038) according to the pretreatment baseline. In propiverine group, the mean value of NCT was significantly reduced by the fourth week (p=0.012). At the twelfth week, NCT had increased and reached approximately pretreatment levels (p=0.438) (Table-2). Temporal Choroidal Thickness: In all three treatment groups (solifenacin, darifenacin, and propiverine), there was no statistically significant difference in the fourth and twelfth weeks when compared to the pretreatment period (p >0.05) (Table-2).

**Table 2:** Measurement results of the sub-foveal, nasal-, and temporal- choroidal thicknesses, intraocular pressure and pupil diameter of the patients initial, 4th and 12th weeks after anticholinergic drug administration.

	Solifenacin (n=70)					Darifenacin (n=55)					Propiverine (n=40)				
	Pretreatment	4th week	p^1^ value	12th week	p^2^ value	Pretreatment	4th week	p^1^ Value	12th week	p^2^ value	Pretreatment	4th week	p^1^ value	12th week	p^2^ value
Subfoveal choroidal thickness (μm) (mean ± SD)	253.52± 26.72	252.84± 26.05	0.215	254.45± 24.97	0.423	258.66± 33.08	259.93± 27.39	0.713	257.68± 21.82	0.748	258.80± 38.08	257.62± 39.13	0.144	258.82± 39.91	0.988
Nasal choroidal thickness (μm) (mean ± SD)	252.48± 24.64	252.87± 24.27	0.042*	252.57± 23.58	0.849	258.70± 23.96	257.51± 22.66	0.002*	255.36± 19.69	0.038*	256.17± 33.34	254.72± 32.81	0.012*	256.95± 33.91	0.438
Temporal choroidal thickness (μm) (mean ± SD)	251.30± 29.43	250.74± 29.70	0.429	249.80± 28.45	0.133	258.08± 22.98	257.49± 22.01	0.218	254.87± 16.59	0.138	251.47± 33.53	251.93± 32.27	0.186	252.41± 34.37	0.490
Intraocular pressure (mmHg) (mean ± SD)	17.30± 2.72	16.67± 2.56	0.006*	16.57± 2.41	0.002*	17.05± 3.40	17.87± 3.24	0.007*	17.53± 3.09	0.097	15.50± 3.02	16.06± 3.00	0.050	15.78± 3.23	0.224
Pupil diameter (mm) (mean ± SD)	4.06± 0.53	4.06± 0.50	0.501	4.07± 0.50	0.329	4.03± 0.43	4.04± 0.45	0.609	4.04± 0.43	0.693	4.04± 0.48	4.08± 0.44	0.009*	4.09± 0.45	0.001*

p^1^: The p value of the statistical difference between the initial and end of the 4th week. (Two sample paired t-test was used)

p^2^: The p value of the statistical difference between the initial and end of the 12th week. (Two sample paired t-test was used)

* p<0.05 value was accepted for the statistical difference.

### Intraocular Pressure

Four and twelve weeks after solifenacin treatment, when compared to the mean level of pretreatment, the mean values of IOP were significantly reduced (p=0.006 and p=0.002, respectively). The results at four weeks after darifenacin treatment showed a significant increase in mean IOP when compared to the mean pretreatment level (p=0.007). The mean IOP level in patients treated with darifenacin after twelve weeks was lower than after the four-week treatment period, and it also did not have any statistically significant difference when compared with the pretreatment IOP levels (p=0.097). In propiverine group, the IOP changes were not found to be statistically significant when compared to the pretreatment levels at forth and twelfth weeks (p >0.05) (Table-2).

### Pupil Diameter

The changes of PD with solifenacin and darifenacin, according to pretreatment levels, were not significantly different at fourth and twelfth weeks (p >0.05). Unlike solifenacin and darifenacin, propiverine treatment significantly increased PD at the fourth (p=0.009) and twelfth weeks (p=0.001) when compared to pretreatment levels (Table-2).

## DISCUSSION

M2 receptors are the most intensive muscarinic receptor types in the bladder and mostly M2 and M3 receptors play a role in OAB physiopathology (21). It is known that darifenacin and solifenacin are more selective for M3 than M2 receptors (6). Propiverine, oxybutynin, fesoterodine, and trospium are nonselective anticholinergics (22, 23). It has also been reported propiverine serves as a calcium channel blocker and increases relaxation in smooth muscles, making it more efficient in treating symptoms of OAB (23). In studies conducted by evaluating the effects of systemic anticholinergics on eyes, it has been emphasized that attention should be paid to the use of these medicines in patients with ophthalmologic diseases, especially dry eye and angle-closure glaucoma (24, 25). We considered these suggestions when choosing criteria for patient’s inclusion in the study.

Telek et al. studied 61 OAB patients and the effects of three months of 1x1 daily oral administration of sustained-release tolterodine on SFCT, NCT, TCT, IOP, and PD (7). When the values before and after treatment and the p-values were compared, it was observed that SFCT (p=0.862), NCT (p=0.658), TCT (p=0.497), IOP (p=0.732), PD (p=0.711) had no differences. The results demonstrated that there was no statistically significant difference on the parameters before and after treatment. However, it was stated that these findings should be supported by other studies.

Turkoglu et al. evaluated daily 1x1 administration of oral trospium to 80 female OAB patients and IOP changes before treatment as well as in the fourth and twelfth weeks of treatment. No significant difference was found in the study for IOP changes in the fourth and twelfth weeks compared to pretreatment (p=0.251, p=0.340, respectively), however, it was reported that trospium significantly reduced tear secretion (26). Likewise, in another study conducted by Turkoglu et al. in 2015, no significant difference was observed in IOP values at commencement, the fourth week, or the twelfth week of daily 1x1 oral solifenacin in 93 female OAB patients (27). It was also emphasized in this study that solifenacin did not affect tear secretion. Sekeroglu et al. compared the IOPs of 60 female OAB patients who used solifenacin for four weeks and a control group of 30 healthy females at commencement and four weeks (28). They found no significant difference in either group (p=0.864 and p=0.160, respectively), and, hence, they reported solifenacin does not affect IOP. In these two studies, effect of solifenacin on CT, IOP, and PD is not statistically significant. However, in these studies, it was observed that solifenacin decreased IOP even though it was not statistically significant in some individuals. In our study, we concluded that solifenacin significantly decreased IOP at the fourth week, and this decline was more pronounced in the twelfth week. Even though IOP significantly decreased in those who used solifenacin, it remained within normal limits (9-22mmHg) in our study.

Altan-Yaycioglu et al. conducted a study on 52 OAB patients, administering oral tolterodine to 28 of them and oxybutynin to remaining 24. The authors then compared the patient’s IOPs and PDs with the initial values at the four-week follow-up. No significant difference was found among the initial values except for PD in dark light in the group that used tolterodine (3.72 vs. 4.16mm, p=0.025) (29). In this study, neither agent affected tear secretion.

Although it is generally recommended that anticholinergics should not be used in OAB patients with angle-closure glaucoma, it was concluded by a randomized, double-blind, placebo-controlled study conducted by Gatchev et al. in 2016 that propiverine does not change IOP in patients with angle-closure glaucoma (30). This study suggested that propiverine could increase PD in patients with wide-angle and angle-closure glaucoma compared to a placebo (28). In our study, we concluded that propiverine did not alter IOP and significantly increased PD at the fourth week and twelfth week.

An important finding of our study is the effect of darifenacin on NCT. According to the results we obtained, usage of darifenacin for twelve weeks progressively decreases NCT. It is reported that the perfusion pressure is important for blood supply to this area (31). Therefore, factors that might spoil ocular blood flow may lead to decreased NCT (32). However, the reason for this finding should be investigated in more detail.

Furthermore, there was a statistically significant difference in the fourth week, and some parameters that improved in the twelfth week also attracted attention in our study. NCT significantly increased and then returned to normal levels for those who used solifenacin, IOP significantly increased and then returned to normal levels for those who used darifenacin, and NCT significantly decreased and then returned to normal levels for those who used propiverine. Perhaps this situation may be related to some adaptation mechanisms in the eye.

This work has several limitations. A major lack of this study is its non-randomized design and the fact that the groups were not evenly distributed. Additionally, even if some baseline characteristics like CT and PD of treatments groups were homogenously distributed, pretreatment IOP from three groups was statistically different. Another notable limitation, anticholinergic-related complications like dry mouth, constipation, and confusion were not mentioned in this cohort. Further works are needed to evaluate the anticholinergic-related complications in collaboration with some parameters for eye and to compare the treatment groups, which have similar baseline characteristics.

In conclusion, solifenacin significantly reduced IOP, darifenacin significantly reduced NCT and propiverine significantly increased PD in patients with OAB who had normal ophthalmologic examinations after the twelve weeks of treatment. These findings can help to decide appropriate anticholinergic drug choice in OAB medical treatment for patients with eye-related disorders. We suggest these findings should be supported by further well-designed randomized prospective studies with larger populations for better reliability.
